# Supplementary material for: The phased pan-genome of tetraploid European potato
Source: Nature. 2025 Apr 16;642(8067):389–97. doi: 10.1038/s41586-025-08843-0 (PMC12158759; doi:10.1038/s41586-025-08843-0)
Supplement: Supplementary file 5 — Supplementary Figs. 3–14, 20, 21, 23–27 and 34–44. [file 41586_2025_8843_MOESM5_ESM.zip › suppl_figure_34-44/suppl_figure_40_G_v3.pdf]

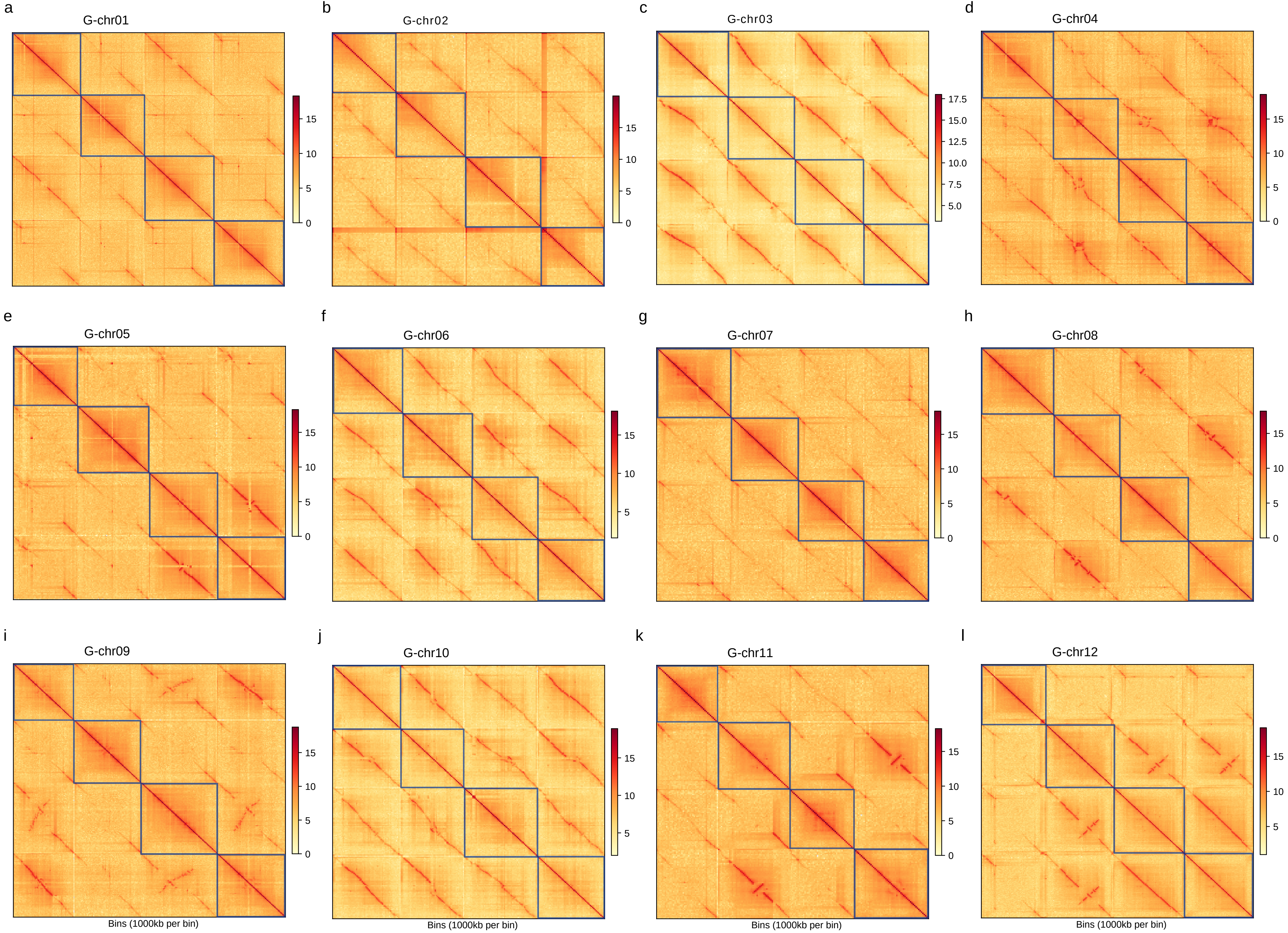

a
G-chr01
b
G-chr02
c
G-chr03
d
G-chr04
e
G-chr05
f
G-chr06
g
G-chr07
h
G-chr08
i
G-chr09
j
G-chr10
k
G-chr11
l
G-chr12
0
5
10
15
5.0
7.5
10.0
12.5
15.0
17.5
Bins (1000kb per bin)


Fla
